# Unsupervised extraction of epidemic syndromes from participatory influenza surveillance self-reported symptoms

**DOI:** 10.1371/journal.pcbi.1006173

**Published:** 2019-04-08

**Authors:** Kyriaki Kalimeri, Matteo Delfino, Ciro Cattuto, Daniela Perrotta, Vittoria Colizza, Caroline Guerrisi, Clement Turbelin, Jim Duggan, John Edmunds, Chinelo Obi, Richard Pebody, Ana O. Franco, Yamir Moreno, Sandro Meloni, Carl Koppeschaar, Charlotte Kjelsø, Ricardo Mexia, Daniela Paolotti

**Affiliations:** 1 ISI Foundation, Turin, Italy; 2 INSERM, Sorbonne Université, Institut Pierre Louis d’Epidémiologie et de Santé Publique, IPLESP, Paris, France; 3 School of Computer Science, National University of Ireland Galway, Galway, Ireland; 4 Department of Infectious Disease Epidemiology, London School of Hygiene and Tropical Medicine, London, United Kingdom; 5 Immunisation and Countermeasures Division, National Infections Service, Public Health England, London, United Kingdom; 6 Instituto Gulbenkian de Ciência, Oeiras, Portugal; 7 Institute for Biocomputation and Physics of Complex Systems (BIFI), University of Zaragoza, Zaragoza, Spain; 8 Science in Action BV, Amsterdam, The Netherlands; 9 Statens Serum Institut, Copenhagen, Denmark; 10 Departamento de Epidemiologia, Instituto Nacional de Saúde Doutor Ricardo Jorge, Lisbon, Portugal; 11 IFISC, Institute for Cross-Disciplinary Physics and Complex Systems (CSIC-UIB), Palma de Mallorca, Spain; 12 Department of Theoretical Physics, University of Zaragoza, Zaragoza, Spain; 13 Sorbonne Université, INSERM, Institut Pierre Louis d’Epidémiologie et de Santé Publique, IPLESP, Paris, France; Northeastern University, UNITED STATES

## Abstract

Seasonal influenza surveillance is usually carried out by sentinel general practitioners (GPs) who compile weekly reports based on the number of influenza-like illness (ILI) clinical cases observed among visited patients. This traditional practice for surveillance generally presents several issues, such as a delay of one week or more in releasing reports, population biases in the health-seeking behaviour, and the lack of a common definition of ILI case. On the other hand, the availability of novel data streams has recently led to the emergence of non-traditional approaches for disease surveillance that can alleviate these issues. In Europe, a participatory web-based surveillance system called Influenzanet represents a powerful tool for monitoring seasonal influenza epidemics thanks to aid of self-selected volunteers from the general population who monitor and report their health status through Internet-based surveys, thus allowing a real-time estimate of the level of influenza circulating in the population. In this work, we propose an unsupervised probabilistic framework that combines time series analysis of self-reported symptoms collected by the Influenzanet platforms and performs an algorithmic detection of groups of symptoms, called *syndromes*. The aim of this study is to show that participatory web-based surveillance systems are capable of detecting the temporal trends of influenza-like illness even without relying on a specific case definition. The methodology was applied to data collected by Influenzanet platforms over the course of six influenza seasons, from 2011-2012 to 2016-2017, with an average of 34,000 participants per season. Results show that our framework is capable of selecting temporal trends of syndromes that closely follow the ILI incidence rates reported by the traditional surveillance systems in the various countries (Pearson correlations ranging from 0.69 for Italy to 0.88 for the Netherlands, with the sole exception of Ireland with a correlation of 0.38). The proposed framework was able to forecast quite accurately the ILI trend of the forthcoming influenza season (2016-2017) based only on the available information of the previous years (2011-2016). Furthermore, to broaden the scope of our approach, we applied it both in a forecasting fashion to predict the ILI trend of the 2016-2017 influenza season (Pearson correlations ranging from 0.60 for Ireland and UK, and 0.85 for the Netherlands) and also to detect gastrointestinal syndrome in France (Pearson correlation of 0.66). The final result is a near-real-time flexible surveillance framework not constrained by any specific case definition and capable of capturing the heterogeneity in symptoms circulation during influenza epidemics in the various European countries.

## Author summary

This study suggests how web-based surveillance data can provide an epidemiological signal capable of detecting the temporal trends of influenza-like illness without relying on a specific case definition. The proposed framework was able to forecast quite accurately the ILI trend of the forthcoming influenza season based only on the available information of the previous years. Moreover, to broaden the scope of our approach, we applied it to the detection of gastrointestinal syndromes. We evaluated the approach against the traditional surveillance data and despite the limited amount of data, the gastrointestinal trend was successfully detected. The result is a near-real-time flexible surveillance and prediction tool that is not constrained by any disease case definition.

## Introduction

Seasonal influenza is an acute contagious respiratory illness caused by viruses that can be easily transmitted from person to person. Influenza viruses circulate worldwide causing annual epidemics with the highest activity during winter seasons in temperate regions and produce an estimated annual attack rate of 3 to 5 million cases of severe illness and about 250 to 500 thousand deaths around the world [1]. National surveillance systems monitor the influenza activity through a network of general practitioners (GPs) who report the weekly number of influenza-like illness (ILI) cases among the overall patients [2]. These traditional surveillance systems usually represent the primary source of information for healthcare officials and policymakers for monitoring influenza epidemics. However, due to the lack of specificity of influenza symptoms, they adopt quantitative indicators (influenza-like illness (ILI) or acute respiratory illness (ARI) being the two most common) which are defined at country level, while no defined standard exists at the international level [3–5]. One main reason might be that classification of ILI cases in GPs’ reports is usually based on common clinical symptoms observed among patients and, as with any syndromic-based disease surveillance, case definitions of “influenza-like illness” can vary [6–10]. They typically include fever, cough, sore throat, headache, muscle aches, nasal congestion, and weakness. Some previous works from hospital-based studies [11, 12], age-specific antiviral trials [7, 13, 14] and national surveillance activities [15] aimed at exploring suitable ILI case symptomatic descriptions but, so far, no unique definition has been widely adopted by the various national surveillance systems worldwide. For this reason, seasonal influenza surveillance in European countries remains rather fragmented. Only in recent years, some state members have adopted the case definition provided by the European Center for Disease Control and Prevention (ECDC) which defines an ILI case as the sudden onset of symptoms with one or more systemic symptoms (fever or feverishness, malaise, headache, myalgia) plus one or more respiratory symptoms (cough, sore throat, shortness of breath) [16]. Nevertheless, a significant fraction of European countries still adopts their own clinical case definition to compile seasonal influenza surveillance weekly reports. S2 Table highlights the existing issue in the heterogeneity of the ILI case definition in Europe [16–18].

In general, differences in seasonal influenza epidemics across European countries are characterised by heterogeneity in sentinel systems, climatic conditions, human mobility systems, as well as social contacts [19, 20]. The result is a consequent heterogeneity in the prevalence of the disease among the population in the various countries which can present differences in severity during the same influenza season. This diversity makes it hard to have a unified, one-fits-all approach to influenza surveillance, let alone a unified ILI definition. Moreover, the ILI definition might change over time even for national sentinel systems [21]. For example, in Italy, the National Institute of Health (Istituto Superiore di Sanità) adopted the ECDC definition only in 2014 [22]. France is a peculiar example as it had double surveillance (ILI and ARI) up till 2014 (Casalegno et al. [3] assessed the performance of various influenza case definitions in France between 2009-2014). Mandl et al. [2] explicitly addressed the variation in the definition of ILI over time.

In recent years the availability of novel digital data streams has given rise to a variety of non-traditional approaches for monitoring seasonal influenza epidemics [23–25]. Such new digital data sources can be exploited to capture additional surveillance signals that can be used to complement GPs surveillance data [26–29]. In this context, some so-called participatory surveillance systems have emerged in several countries around the world with the aim of monitoring influenza circulation through Internet reporting of self-selected participants [30–32]. One of these systems, the Influenzanet project [30], has been established in Europe since 2011 and it is now present in ten European countries. In this study, we excluded from the analysis the country of Sweden, due to the fact that the Swedish cohort is solicited upon invitation when required and not on an annual basis [33]. The system relies on the voluntary participation of the general population through a dedicated national website in each country involved in the project. Data are obtained on a weekly basis through an online survey [34] where participants are invited to report whether they experienced or not any of the following symptoms since their last survey: fever, chills, runny or blocked nose, sneezing, sore throat, cough, shortness of breath, headache, muscle/joint pain, chest pain, feeling tired or exhausted, loss of appetite, coloured sputum/phlegm, watery/bloodshot eyes, nausea, vomiting, diarrhoea, stomach ache, or other symptoms. Differently, from most traditional surveillance systems, this participatory form of online surveillance allows the collection of symptoms in real-time and directly from the general population, including those individuals who do not seek health care assistance. The list of proposed symptoms has been chosen to include the various ILI definitions adopted by national surveillance systems in Europe and, at the same time, to get a comprehensive list of symptoms that could be clearly articulated and understood by participants and would allow the detection of various circulating flu-related illnesses. Even though participatory systems generally suffer from self-selection biases, causing the sample to be non-representative of the general population [35], previous works have shown that the web-based surveillance data collected by Influenzanet can provide relevant information to estimate age-specific influenza attack rates [36, 37], influenza vaccine effectiveness [34, 38, 39], risk factors for ILI [39–41], and to assess health care seeking behaviour [39, 42]. Moreover, it has been largely demonstrated that weekly ILI incidence rates computed from the web-based surveillance data by applying the ECDC case definition to the set of self-reported symptoms correlate well with the weekly ILI incidence reported by GPs surveillance [37, 39, 43].

An additional advantage of collecting symptoms directly from individuals among the general population in the various Influenzanet countries is that it is straightforward to compare the prevalence and the temporal dynamics of specific symptoms or groups of symptoms from one country to the other. In a previous work focused on France [44], the authors proposed population-level indicators based on self-reported symptoms and analysed crowdsourced incidence estimates comparing them to official estimates provided by sentinel systems.

In this work, we propose an approach that aims at addressing the heterogeneity of seasonal influenza epidemiological signals in the various European countries, focusing on the individual symptoms collected directly from the general population. The goal is to develop a mathematical framework able to extract, in an unsupervised fashion, the groups of symptoms that are in good correlation with the ILI incidence, as detected by traditional surveillance systems for each country without imposing an a priori a specific ILI case definition. By using the daily occurrence of symptoms in form of matrix, we employ an approach based on Non-negative Matrix Factorization (NMF) [45], to extract *latent*^1^ features of the matrix that correspond to linear combinations of groups of symptoms. We assume that a specific combination of reported symptoms is the symptomatic expression of one or more illnesses experienced by the participants, i.e. of the *syndromes* affecting the individual. We can then select those groups of symptoms that better correlate with the sentinel-based ILI incidence, which will become our best approximation for the actual influenza-like illness signal for a specific country.

The overall encouraging results suggest that such methodology can be employed as a near real-time flexible surveillance and prediction tool not constrained by any disease case definition. Thus, it can be employed to monitor a wide range of symptomatic infectious diseases or to nowcast the influenza trend, to help to devise public health communication campaigns.

## Materials and methods

### Ethics statement

This study was conducted in agreement with country-specific regulations on privacy and data collection and treatment. Informed consent was obtained from all participants enabling the collection, storage, and treatment of data, and their publication in anonymized, processed, and aggregated forms for scientific purposes. In addition, approvals by Ethical Review Boards or Committees were obtained, where needed according to country-specific regulations. In The United Kingdom, the Flusurvey study was approved by the London School of Hygiene and Tropical Medicine Ethics Committee (Application number 5530). In France, the Grippenet.fr study was approved by the Comité consultatif sur le traitement de l’information en matiére de recherche (CCTIRS, Advisory committee on information processing for research, authorization 11.565) and by the Commission Nationale de l’Informatique et des Libertés (CNIL, French Data Protection Authority, authorization DR-2012-024). In Portugal, the Gripenet project was approved by the National Data Protection Committee and also by the Ethics Committee of the Instituto Gulbenkian de Ciência.

### Data collection

#### Influenzanet

Since the winter season of 2011-2012, the Influenzanet platforms share a common and standardized data collection approach throughout the nine European countries involved, namely: Belgium (BE), Denmark (DK), France (FR), Ireland (IE), Italy (IT), the Netherlands (NL), Portugal (PT), Spain (ES) and the United Kingdom (UK). In each of the Influenzanet countries, the national platform is coordinated by a team of local researchers from Universities, Research Institutions or Public Health Institutions and consists of a website where individuals can register and have access to a personal account where they can insert and update their data. The platforms are disseminated among the general population through press releases, public media campaigns, specific dissemination events (e.g. science fairs) or word of mouth. Participation is voluntary and anonymous, and all the residents of the participating countries can enrol. Upon registration, individuals are asked to complete an online Intake Questionnaire covering basic questions such as age, gender, household size and composition, home location, workplace, etc. [46]. Participants can also create accounts on behalf of other members of their family or household, thus enabling, for instance, parents to record data for their children. Registered participants are then reminded weekly, via an e-mail newsletter, to fill in a Symptoms Questionnaire [46] in which they are presented with a list of general, respiratory and gastrointestinal symptoms (18 in total, reported in Table 1) and asked whether since the last time they visited the platform they experienced any symptoms among those listed. In this study, we employed data collected by the Influenzanet platforms in the nine European countries over the course of six influenza seasons, from 2011-2012 to 2016-2017.

**Table 1:** List of Influenzanet Symptoms.

Fever	Chills	Runny/blocked nose	Sneezing
Sore throat	Cough	Shortness of breath	Headache
Muscle/joint pain	Chest pain	Feeling tired (malaise)	Loss of appetite
Coloured Sputum/Phlegm	Watery, bloodshot eyes	Nausea	Vomiting
Diarrhoea	Stomach ache	Sudden Onset	

List of the 18 symptoms presented to Influenzanet participants in the weekly Symptoms Questionnaire, plus the sudden onset variable, i.e. if symptoms appeared suddenly over a few hours.

#### Traditional ILI surveillance

Seasonal influenza is traditionally monitored by national networks of general practitioners (GPs) who report the weekly number of visited patients with influenza-like illness symptoms according to the national ILI case definition. Despite some practical limitations, mainly due to a heterogeneous population coverage and a considerable delay in disseminating data, such traditional surveillance data are generally considered as ground truth. Therefore, we used the traditional ILI surveillance data to evaluate the performance of our framework developed on the Influenzanet data. In this study, we used the weekly ILI incidence data for 6 influenza seasons, from 2011-2012 to 2016-2017, collected from the ECDC dedicated web page [47] for all countries, except France, for which, instead, we obtained the weekly data on the ILI incidence and gastrointestinal infections directly from the national network, called *Réseau Sentinelles* [48]. All reports were accessed and downloaded in March 2017.

### Data preprocessing

In general, the inclusion criteria of participants in the data analysis vary depending on the specific aim of the study [35, 39, 49, 50]. In our case, we included only the individuals registered on the Influenzanet national platforms who filled in at least one Symptoms Questionnaire (hereafter referred to as “survey”) per season. This was done to focus the analysis on participants for which we have some information. We had to necessarily exclude individuals who have registered on the platforms but who have not submitted any symptoms survey during any influenza season. This corresponds to the exclusion of 0.3% of the registered participants.

Moreover, to reduce the noise due to low participation rates at the beginning of the data collection of each influenza season, we consider as starting point the first week for which the number of surveys corresponded at least to 5% of the total number of the surveys filled during the week with the highest participation for that season. This refers to the fact that at the beginning of the season, which is a period when the epidemic is still well below the epidemic threshold, the participation (i.e. the number of symptoms surveys) is rather low and therefore the signal to noise ratio can be very low too. Furthermore, we included only one survey per each week—the latest one—if more than one survey was submitted during the same week by the same participant. This exclusion corresponds to a small fraction of discarded surveys, approximately 5% of the total number of surveys; moreover, the distribution of the discarded symptoms and the submission time of the dropped surveys, are homogeneous^2^. This exclusion criterion is essential to express the number of self-reported symptoms as probabilities in the final ILI syndrome emerging from our framework and to interpret the aggregation of symptoms as an “incidence”.

S1 Table in the supporting information presents descriptive statistics for each country, namely: (i) the number of seasons analysed, (ii) the average number of participants per season, (iii) the average number of weekly surveys per season, (iv) the average percentage of surveys with at least one symptom, (v) the average number of surveys per participant per season and (vi) the average number of weeks within a single season.

### Temporal syndrome modeling and non-negative matrix factorization

In this section, we describe the methodology employed to extract the latent features from the self-reported symptoms collected by the various Influenzanet platforms of the participating countries. Our approach relies on the assumption that a specific group of self-reported symptoms corresponds to the symptomatic expression of one or more illnesses, hereafter called *syndromes*, circulating among the population sample of Influenzanet. In our study we consider the 18 symptoms presented in the weekly Symptoms Questionnaire plus an additional symptoms-related variable, called “Sudden onset”, referring to the sudden appearance of symptoms, typically over the course of the previous 24 hours (see Table 1). This totalizes 19 symptom variables that we hereafter designate interchangeably as “symptoms”. The symptoms were treated as binary boolean variables having value 1 if the symptom is present and 0 if the symptom is absent. We then aggregated the reported symptoms across all participants to build a matrix **X** = [*x*_*ij*_], whose elements contain the occurrences of each symptom *j* ∈ {1, ‥, *J*} during each day *i* ∈ {1, ‥, *I*}. In other words, each element of the matrix corresponds to the number of times each symptom has been reported on each day of the influenza seasons under study. The result is a high-dimensional sparse matrix that can be linearly decomposed through a Non-negative Matrix Factorization (NMF) technique [45]. We opted for NMF since its non-negativity constraint offers the advantage of a straightforward interpretation of the results as positive quantities that can then be associated with the initial symptoms. This approach can be considered as a “blind source separation” problem [51] in which neither the sources nor the mixing procedure is known, but only the resulting mixed signals are measured.

In our case, the time series corresponding to the daily symptoms counts are measured by the Influenzanet platforms and can be considered as the result of a linear mixing process driven by unknown sources, i.e. the latent syndromes. In the following we will use interchangeably the terms *syndrome*, *source* or *component*. According to this consideration, each element *x*_*ij*_ of the matrix **X** can be expressed as follows:

$$\begin{array}{c} x_{ij}=\sum_{k\in \{1,‥,K\}}w_{ik}\;h_{kj}+e_{ij}, \end{array}$$ (1)

where the coefficients *h*_*kj*_ describe the set of the unknown *K* sources, the factor *w*_*ik*_ represents the time-dependent mixing coefficients, and the terms *e*_*ij*_ correspond to the approximation error. The mixing equations Eq (1) can be equivalently expressed in matrix notation as:

$$\begin{array}{c} X=WH+E \end{array}$$ (2)

where:

$$\begin{array}{c} W=\left[w_{ik}\right]\;,\;H=\left[h_{kj}\right]\;,\;E=\left[e_{ij}\right] \end{array}$$ (3)

It is worth stressing that in this representation the matrix **X** is known, while the matrices **W** and **H** are unknown and determined by the NMF algorithm. In particular, we used a variation of the NMF algorithm that minimizes the Kullback-Leibler divergence loss function [52] defined as follows:

$$\begin{array}{c} \underset{W,H}{\text{argmin}}\sum_{i,j}x_{ij}\text{log}\;(\frac{x_{ij}}{{\hat{x}}_{ij}})-x_{ij}+{\hat{x}}_{ij}, \end{array}$$ (4)

where:

$$\begin{array}{c} {\hat{x}}_{ij}=\sum_{k}w_{ik}h_{kj}. \end{array}$$ (5)

To minimise the Kullback-Leibler divergence loss function, we adopted the multiplicative update rules described in [53]. Note that different initialisation of the matrices **W** and **H** might lead to different local minima, making the interpretation of the results not straightforward. To overcome this issue, we used an initialization technique called Non-negative Double Singular Value Decomposition [54], that is based on a probabilistic approach equivalent to the probabilistic latent semantic analysis (pLSA) [55], employed in the context of semantic analysis of text corpora. Since the two approaches of NMF and pLSA are equivalent (see [56] for more details), the results of our matrix decomposition can be probabilistically interpreted as a mixture of conditionally independent multinomials, that we call *p*(*i*, *j*). We can then write:

$$\begin{array}{c} \begin{array}{cc} \pi (i,j)\approx p(i,j) & =\sum_{k}p\left(k\right)\;p\left(i,j|k\right)\\ & =\sum_{k}p\left(k\right)\;p\left(i|k\right)\;p\left(j|k\right), \end{array} \end{array}$$ (6)

where:

$$\begin{array}{c} \pi \left(i,j\right)=x_{ij}/N,\;N=\sum_{i,j}x_{ij} \end{array}$$ (7)

and *N* is the total number of symptoms counts.

According to Eq (6), the total number of symptoms counts will be proportionally split among *K* latent sources according to *p*(*k*), which is the probability to observe a specific component *k*; *p*(*i*|*k*) is the probability to observe a component *k* in a day *i* and *p*(*j*|*k*) is the probability to observe a specific symptom *j* in a component *k*, and they can be expressed as follows:

$$\begin{array}{c} \begin{array}{cccccccccc} & p(i|k) & =\; & w_{ik}/\sum_{i}w_{ik} & \; & , & \; & \sum_{i}p\left(i|k\right) & = & 1,\\ & p(j|k) & =\; & h_{kj}/\sum_{j}h_{kj} & \; & , & \; & \sum_{j}p\left(j|k\right) & = & 1,\\ & p(k) & =\; & \sum_{i}w_{ik}\;\sum_{j}h_{jk}/N & \; & , & \; & \sum_{k}p\left(k\right) & = & 1. \end{array} \end{array}$$ (8)

At this point, Eq (8) allows to determine the probability *p*(*i*, *k*) that, rescaled on the total number of symptoms counts *N*, yields the desired decomposition procedure, *y*_*ik*_, which represents the contribution of a specific component *k* in a day *i*, given by the following expression:

$$\begin{array}{c} y_{ik}=N\;p\left(i,k\right)=N\;p\left(k\right)\;p\left(i|k\right) \end{array}$$ (9)

Thus, the final step in our approach is to determine the optimal number of components *k*_*min*_ to be used for the decomposition. A natural upper bound for *k* would be the total number of symptoms, i.e. 19. We need to determine the number of components with the best trade-off between a model that best approximates the original matrix *X* and at the same time does not overfit the data. Each time we minimize the loss function Eq (4) for a specific number of components *k*, we obtain a candidate decomposition. To determine the best decomposition, we use an approximated model selection criterion, known as the Akaike Information Criterion (*AIC*) [57]. In particular, we employ the corrected version of the Akaike Information Criterion (*AIC*_*c*_) proposed in [58], valid for finite sample sizes. For each of the candidate decompositions generated by the various values of *k*, we estimate the value of *AIC*_*c*_(*k*), expressed as:

$$\begin{array}{c} {AIC}_{c}\left(k\right)=-2L\left(k\right)+2P+2\frac{P(P+1)}{N-P-1}, \end{array}$$ (10)

where *L*(*k*) is the log-likelihood of the model with *k* components, defined in [56] as:

$$\begin{array}{c} L\left(k\right)=\sum_{i,j}x_{ij}\text{log}\;p\left(i,j\right). \end{array}$$ (11)

*P* is the number of parameters of the model defined as:

$$\begin{array}{c} P=K\;(I+J-2)-1, \end{array}$$ (12)

where *K* is the upper bound for the number of components, *I* is the total number of days and *J* is the total number of symptoms. The best candidate decomposition is the one that minimizes Eq (10) and we denote it as *AIC*_*c*_(*k*_*min*_). The final result is a model, that we call $y_{ik_{min}}$, consisting of *k*_*min*_ components that best approximate the original matrix *X*.

### Data analysis

We applied the aforementioned framework to the data collected by the Influenzanet platforms in nine European countries throughout six influenza seasons (from 2011-2012 to 2016-2017). For each country, we applied the decomposition algorithm to the symptoms’ matrix **X** as represented in Eq (2) and, based on the AIC, we obtained the “optimum” number of components, *k*_*min*_, for the decomposition. The daily counts of the emerged components are eventually aggregated weekly to allow the comparison with the weekly incidence reported by the traditional GPs surveillance. Among the *k*_*min*_ latent components, i.e. syndromes, extracted for each country, we identified the one that correlates better with the time series reported by the traditional GPs surveillance. In the following, we denote this component as IN_NMF. This component corresponds to the combination of symptoms that more closely represent the ILI time series recorded by the traditional surveillance, and hence, it can be used to build a data-driven, unsupervised ILI case definition, which is the ultimate goal of this study.

To further evaluate the IN_NMF signal selected for each country, we also computed the Pearson correlation between: (i) the IN_NMF and the time series obtained by applying the ECDC case definition to the Influenzanet data (hereafter called IN_ECDC); (ii) the IN_NMF and the ILI incidence reported by the national surveillance systems per country (hereafter called GP); and (iii) the IN_ECDC and the GP. The reported correlations refer to the time series over the entire period analysed (2011-2017).

Additionally, we explored the predictive power of the proposed methodology in the following way: first, we trained the NMF decomposition framework with Influenzanet data only from 2011 to 2016 and then, we employed the resulting symptom weights to infer the weekly IN_NMF estimates during the 2016-2017 season. To assess the quality of this signal, we evaluated the Pearson correlation of the forecasted IN_NMF time series for 2016-2017 with both the GP time series and the IN_ECDC time series.

Moreover, to broaden the scope of our framework in identifying syndromes not related to ILI (e.g. gastrointestinal *versus* respiratory), we employed it to identify the syndrome related to gastrointestinal episodes by performing the Pearson correlation with data provided by the traditional official surveillance in France. We focused on the case of France due to the immediate data availability from the official surveillance. The *Réseau Sentinelles* in fact comprises a unique program of data collection about gastrointestinal illness episodes [59]. The identified component is denoted as IN_Gastro. For the entire analysis and simulations we used the Python programming language (Python Software Foundation, version 2.7, https://www.python.org/).

## Results

### ILI selection of components

S1 Fig in the supporting information depicts an exploration on the relative AIC values of a series of candidate models (*AIC*_*c*_(*k*) − *AIC*_*c*_(*k*_*min*_), with *k* ∈ [1, 6]), estimated according to Eq (10). For the majority of the countries, the optimal decomposition consisted of *k*_*min*_ = 2 components, with the exceptions of the Netherlands and Belgium with *k*_*min*_ = 3, and France with *k*_*min*_ = 4. S2, S3, S4, and S5 Figs in the supporting information depict for each country the respective time series of all the emerging *k*_*min*_ components and their symptoms composition. The component selected by our framework is highlighted by a blue square. These results show how our approach is capable of taking into account differences in ILI definition between countries since we can select the components that best correlate with the national ILI signal.

### ILI component analysis

**Fig 1:**
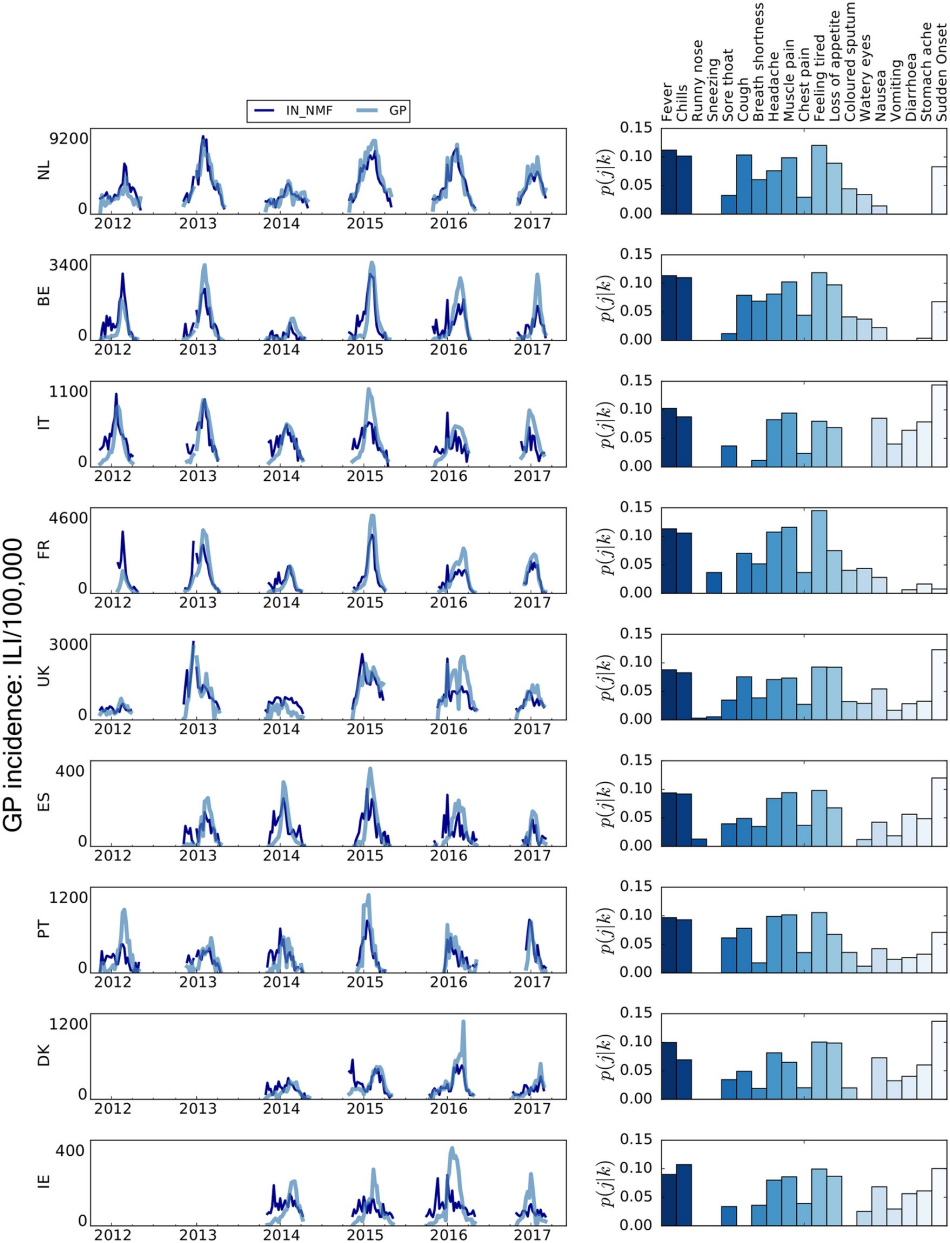
Qualitative comparison between the IN_NMF and the national surveillance ILI incidence (GP) time series and IN_NMF component composition. Left panel: qualitative comparison between the IN_NMF and the national surveillance ILI incidence (GP) time series. To allow for easier visual inspection, the depicted IN_NMF syndromes are rescaled by a fixed factor to the respective GP incidence. On the y-axis, the sample size of the GP incidence is reported. Right panel: contribution of each symptom to the automatically selected IN_NMF component. The bars are coloured for readability purposes only.

In the left panel of Fig 1, the IN_NMF component for each country is shown in comparison to the ILI signal as recorded by the traditional surveillance, GP. To allow for visual comparison, the IN_NMF time series has been rescaled on the GP time series with a fixed scaling factor. Specifically, the IN_NMF has been rescaled on the highest peak among all the GP time series for each country, hence the lower peak of the IN_NMF for the other peaks of the GP time series. Consequently, the performance of the selected ILI component cannot be evaluated in terms of amplitude and error with respect to the peak estimate.

In the right panel of Fig 1, the break-down of symptoms for each country’s IN_NMF component is expressed in terms of probabilistic contributions, denoted as *p*(*j*|*k*), as described in Eq (6). In terms of symptoms’ composition, IN_NMF appears to be stable across the various countries and consistent with the expected set of symptoms clinically associated with ILI. The top contributing symptoms are fever, chills and feeling tired, often reported in combination with a sudden onset of symptoms. Notably, each of these top three symptoms contributes for about 10% or more of the overall component composition. This is consistent across all the nine countries and it is the most important result of this study since it represents the basis towards the development of a common ILI definition. Small heterogeneities in the component composition across countries are most likely due to differences in the ILI case definitions used by sentinel doctors in each country which are reflected in the data that we use as ground truth. In principle, this issue might be overcome by using seroprevalence data as ground truth.

For the sake of comparison, we have examined how our framework performs with respect to other similar approaches. For example, Goldstein et al. [60] have used two inference methods to estimate incidence curves from symptoms surveillance data. The first method essentially assumes that the distribution of symptoms is known. In our case, we have no such assumption; instead, we extract the symptoms and their probabilistic distribution from the observed data without making any a priori assumption on the distribution of symptoms. The second inference method proposed by Goldstein et al. [60] is closer to our framework and falls under the umbrella of the term “blind source separation”. The Non-negative Matrix Factorization can be formulated as an expectation-maximization problem [61]. The difference with our approach is that they assume as an initial condition that the expected weekly incidence is equal to 1 for each infection in their survey sample. Their approach is sensitive to the ratio of flu/non-flu distribution while NMF manages to overcome this problem.

### ILI model evaluation

Table 2 reports all the Pearson correlations between the different time series as mentioned in the Data Analysis section. For all countries, the correlation between the IN_NMF component and the IN_ECDC is very high, ranging from 0.82 to 0.92 (row (i)), thus showing that the IN_NMF signal captures symptoms highly compatible with those present in the ECDC ILI definition applied to the Influenzanet data. However, by carefully examining rows (ii) and (iii), we note slight variations per country. For the Netherlands, Belgium, and Ireland the ILI incidence reported by the traditional surveillance (GP) was more strongly correlated with the IN_NMF component, than with the ILI incidence obtained by applying the ECDC ILI definition to the Influenzanet data (IN_ECDC). For the United Kingdom, Spain, Denmark, and Portugal, the IN_NMF components perform equally well as the IN_ECDC. For Italy and France, the IN_NMF component had a slightly lower correlation (about 11% and 7% less respectively) with the traditional surveillance data (GP) than the IN_ECDC. Ireland is the only country for which we obtain a low correlation between the traditional surveillance data (GP) and both the IN_NMF and IN_ECDC, probably due to the limited number of participants in Influenzanet (see S1 Table in the supporting information). Despite this, the IN_NMF performs much better than the IN_ECDC in capturing the ILI incidence trend in Ireland (0.38 vs 0.23). This variation in performance is not an issue for the goal of this work since our focus is on paving the way towards a common cross-country ILI definition rather than finding the perfect signal that correlates best with the traditional national surveillance. Also, the loss in performance of IN_NMF vs GP with respect to IN_ECDC vs GP for Italy and France is only a small percentage. One might argue that, since it has been observed that people tend to go to the doctor if their symptoms are more severe or if the duration of the disease is longer [62], the high correlation between the IN_NMF time series and the GP time series might be attributable to the fact that participatory surveillance only captures individuals with perceived severe symptoms, who did visit a doctor for their illness. Unfortunately, we cannot assess the severity of self-reported symptoms, but we can assess the fraction of participants who claimed they have visited a healthcare provider for their symptoms and, in line with previous studies, we found that the vast majority of participants did not seek medical consultation. Specifically, the percentages of participants who did seek medical consultation per country are: NL 12%, BE 22%, IT 23%, FR 26%, UK 14%, ES 17%, PT 17%, DK 11%, IE 16%.

**Table 2:** Pearson correlations with the ground-truth data per country.

	NL	BE	IT	FR	UK	ES	PT	DK	IE
(i) IN_NMF vs IN_ECDC for the seasons 2011-2017									
	0.91	0.92	0.86	0.83	0.92	0.86	0.84	0.90	0.82
(ii) IN_NMF vs GP for the seasons 2011-2017									
	0.88	0.80	0.69	0.79	0.74	0.65	0.66	0.71	0.38
(iii) IN_ECDC vs GP for the seasons 2011-2017									
	0.79	0.72	0.80	0.86	0.75	0.67	0.63	0.68	0.23
(iv) IN_NMF forecast vs GP for the season 2016-2017									
	0.85	0.82	0.69	0.80	0.60	0.84	0.80	0.76	0.60
(v) IN_NMF forecast vs IN_ECDC for the season 2016-2017									
	0.85	0.82	0.86	0.93	0.67	0.59	0.88	0.80	0.71

(i) Pearson correlation between the time series of IN_NMF with the respective time series produced when applying the ILI definition on the Influenzanet data (IN_ECDC). (ii) Pearson correlation between IN_NMF and the respective ILI incidence reported by the national surveillance systems per country (GP). (iii) Pearson correlation between ILI incidence obtained by applying the ECDC case definition to raw Influenzanet data (IN_ECDC) and ILI incidence reported by the national surveillance systems per country (GP). (iv) Pearson correlation between the forecasted 2016-2017 IN_NMF and ILI incidence reported by the national surveillance systems per country (GP) for the season 2016-2017. (v) Pearson correlation between ILI incidence obtained by applying the ECDC case definition to raw Influenzanet data (IN_ECDC) and the respective forecasted IN_NMF for the 2016-2017. Note that the reported correlations are not averages per ILI seasons per country but the correlation of the time series of the entire period (2011-2017 for (i),(ii) and (iii) and 2016-2017 for (iv) and (v)) between the IN_NMF and the respective GP time series for each country.

Moreover, to investigate the performance of our framework with respect to healthcare seeking behaviour, we employed two different approaches. First, we trained our framework only with the subset of self-reported symptoms from participants who consulted a medical doctor for their symptoms, obtaining the following Pearson correlations with the GP time series: NL 0.83, BE 0.82, IT 0.87, FR 0.92, UK 0.88, ES 0.82, PT 0.82, DK 0.69, IE 0.51. Secondly, we trained our framework only with the subset of self-reported symptoms from participants who did not consult a medical doctor for their symptoms, obtaining the following Pearson correlations: NL 0.77, BE 0.59, IT 0.69, FR 0.78, UK 0.72, ES 0.54, PT 0.48, DK 0.64, IE 0.29. We notice that since by default our framework selects as ILI component the one that best correlates with the official surveillance, the IN_NMF signal emerged represents better the data reported by the official surveillance systems. Unsurprisingly, the correlations are higher when we compare the same population of individuals who did seek medical consultation for their illness. On the other hand, it is of extreme importance that our framework is capable of extracting a relevant signal in the latter case since the population of individuals who do not seek healthcare is complementary to the one depicted by the official surveillance data.

Finally, in order to assess the impact of the exclusion criterion for which we do not take into account duplicate reports from the same individual in a single week, we have determined the mean percentage of the symptoms discarded per country: NL 0.04%, BE 0.03%, IT 0.09%, FR 0.06%, UK 0.16%, ES 0.05%, PT 0.12%, DK 0.03%, IE 0.10%. Indeed, the duplicate report exclusion corresponds to a small number of symptoms discarded each week and the distribution of all discarded symptoms is homogeneous.

### ILI prediction evaluation

The results of the prediction analysis described in the Data Analysis section are shown in S6 Fig. The fourth row of Table 2 (iv) reports the correlations of the forecasted IN_NMF time series and the national surveillance for the season 2016-2017 (GP). The correlation between the two time series is good for all the countries, ranging from 0.60 to 0.85. In supplementary information we depict the results of the prediction analysis described in the Data Analysis section. As already stated above, for the sake of visual comparison, the IN_NMF time series has been rescaled to the highest peak of the GP time series for each country, hence the lower peak for the other peaks. Consequently, the two time series cannot be evaluated in terms of amplitude and error.

In Table 2 row (v), we also report the correlation between the forecasted IN_NMF time series and the IN_ECDC time series emerged from applying the ECDC definition to the Influenzanet data for the season (2016-2017). Also, in this case, the predicted trend of the ILI component have high correlations, ranging from 0.59 to 0.93.

Even if the focus of the paper is on the possibility of extracting a symptoms-based data-driven definition of ILI that is country specific, the forecasting capabilities of the framework represent an additional strengthening factor (the forecasting potential of using participatory surveillance data, in combination with additional epidemiological signals has also been explored in a previous paper [29]). To further assess the robustness of the forecasts produced by the NMF framework, we have compared their accuracy with respect to a null model in two different ways.

1. We trained a model following our NMF framework on the shuffled counts of symptoms observed among the users during the seasons of 2011-2016. Then, the resulting model was employed to infer the IN_NMF trend of the Influenzanet data collected in 2016-2017. Despite being trained on randomly shuffled data, the selected ILI component correlates well with the incidence estimated by sentinel doctors, but the combinations of symptoms in the syndrome are rather inconsistent (see S7 Fig in Supporting Information). Pearson correlations per country are: BE 0.81, DK 0.76, ES 0.74, FR 0.91, IE 0.66, IT 0.85, NL 0.85, PT 0.88, UK 0.61.
2. We trained a model following our NMF framework on the data from seasons 2011-2012 to 2015-2016, and then, we used it to predict the ILI component of season 2016-2017, randomly shuffling the resulting symptoms. In this case, the Pearson correlations are extremely low: BE 0.25, DK 0.08, ES -0.04, FR 0.25, IE 0.03, IT -0.15, NL 0.30, PT 0.07, UK 0.13. This provides us with a measure of how worse our predictions become with a random combination of symptoms.

### Gastrointestinal component evaluation

**Fig 2:**
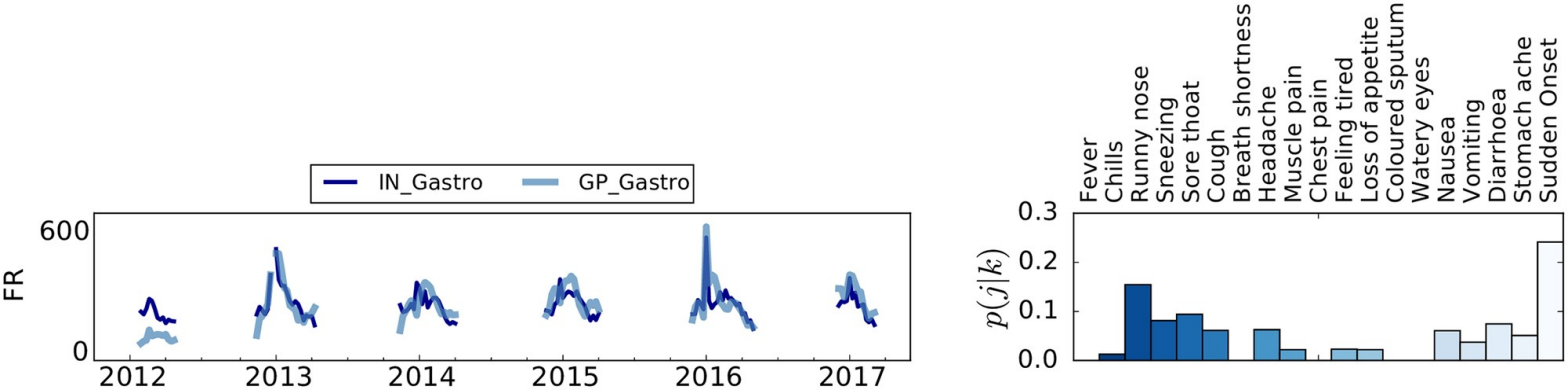
Composition of the IN_Gastro component and comparison with the incidence of acute diarrhoea detected by the national surveillance data (GP_Gastro) for France. Left panel: Time series comparison between the IN_Gastro component and the incidence of acute diarrhoea detected by the national surveillance data (GP_Gastro) for France. To allow for an easier visual inspection the depicted IN_Gastro syndrome is rescaled by a fixed factor on the respective GP_Gastro incidence. On the y-axis, the sample size of the GP incidence is reported. Right panel: symptomatic contribution of the automatically selected IN_Gastro component. The bars are coloured for readability purposes only.

In the left panel of Fig 2, we show the time series for the incidence of acute diarrhoea episodes (GP_Gastro) as detected by the official national surveillance in France, and the time series of the syndrome identified by our framework (IN_Gastro). The Pearson correlation between the extracted syndrome and the official surveillance data is 0.66.

In the right panel of Fig 2 we depict the probabilistic contribution of each symptom to the IN_Gastro syndrome. Emerging symptoms, in this case, include also stomach ache, diarrhoea, and vomiting, which are in line with our expectations. Even if respiratory symptoms like runny nose or sneezing are also present, the contribution of fever and chills (which were the main contributors to the IN_NMF signal) is almost negligible. This suggests a rather good capability of our framework in discriminating between different syndromes. Despite limitations of the data availability, these preliminary findings indicate that the latent components of the decomposition, not related to ILI, may express syndromes related to allergies, common-cold or gastroenteritis. Understandably, additional adequate surveillance data are required to make a firm statement and reach a robust interpretation of the syndromes.

Previous works have also focused on detecting gastrointestinal symptoms circulating among the general population through digital unstructured data [33, 63–65] from participatory surveillance, big data, such as Twitter, as well as national pharmacy sales data. These examples show how crowdsourced digital health-related data, as well as passive digital traces generated on the web by individuals from the general population, can complement traditional and syndromic surveillance systems to estimate the circulation of gastrointestinal syndromes. This is particularly important because only a fraction (about a third) of individuals who reported gastrointestinal symptoms in France also declared that they visited a doctor. The NMF framework applied to the subset of data from participants who did not visit a doctor for their symptoms selected a component whose correlation with official surveillance data is 0.67 (with respect to a correlation of 0.66 when using all the data). This shows that people tend to visit a doctor rarely and probably only if their symptoms are severe. The NMF framework is capable of providing robust results even if we focus the analysis only on those individuals who did not visit a doctor, for which we can safely assume that their symptoms were not severe.

### Limitations and future work

This approach has several limitations. As far as data are concerned, crowdsourced digital data are intrinsically biased due to the fact that the participants are self-selected and not representative of the general population, as extensively explored in a previous work [35]. However, such sample biases do not affect the robustness and accuracy of the epidemiological signal detected through participatory surveillance [37, 39, 43]. Previous works have shown that selecting groups with specific reporting patterns or combining data sources can improve the representativeness [28, 66, 67]. Extending this study, we will incorporate in our framework the user attributes to account for selection biases.

Other issues could rise from the variable reporting behaviours along the season, individuals’ interpretation of the terms used for surveillance, and the correctness of their self-assessments. Some of these issues have been addressed partially in previous works [10, 40, 44, 50, 68]. In our approach, we assume that self-reported symptoms are consistent since Influenzanet data have been already proven to be accurate and reliable for ILI surveillance, even without providing any clinical confirmation. However, we are aware that one of the criticisms of online participatory surveillance is the lack of virological confirmation of influenza cases that would instead help to better assess the actual circulation of influenza in the population. To this respect, a pilot study has been developed in the United Kingdom by the national Influenzanet platform, called Flusurvey, which demonstrates that self-swabbing can be applied to an online cohort to conduct virological laboratory testing [69].

Moreover, in this work, we have not compared the performance of other machine learning algorithms besides NMF since this would go beyond the scope of this paper. Future work could explore the performance of other methods and clustering algorithms. Among the many algorithmic choices, LDA could be employed in a similar framework, since PLSA is simply a special case of LDA and Faleiros et al. [70] showed that indeed NMF with Kullback-Leibler divergence approximates the latent Dirichlet allocation (LDA) model under a uniform Dirichlet prior distribution.

Finally, there are inherent socio-economic biases in influenza surveillance systems [71] due to the fact that in some countries traditional surveillance is based on primary healthcare which may be biased towards population with higher socioeconomic status. Even additional digital unstructured data sources are more representative of these population groups, thus even combining traditional and non-traditional data sources might fail in mitigating biases towards more at-risk groups.

## Discussion

The practice of seasonal influenza surveillance is affected by a lack of a common case definition for influenza-like illness across countries. Moreover, the seasonal influenza epidemics in the various European countries present a high degree of heterogeneity. To improve seasonal influenza surveillance beyond these issues, we propose an unsupervised probabilistic framework based on self-reported symptoms collected daily through a network of participatory web-based influenza surveillance platforms in Europe called Influenzanet. Our approach, which relies on a Non-negative Matrix Factorization of the daily symptoms matrix, is capable of producing an epidemiological signal that does not rely on a specific a priori case definition and that follows the temporal trend of influenza-like illness closely as detected by the traditional sentinel doctors surveillance in each country. The emerging signal successfully captures the ILI incidence trend estimated by the national surveillance data for all the nine countries included in this study. We also demonstrate that the proposed approach can be employed to forecast the forthcoming ILI incidence. Additionally, the proposed approach has the potential to be used to identify other illnesses, as shown here for gastrointestinal syndromes, although additional traditional surveillance data is needed to validate the generalisability of our framework. We can thus conclude that there is great potential in using symptoms directly collected from the general population to inform unsupervised algorithmic approaches aimed at detecting circulating bouts of illnesses without imposing an a priori case definition. The standardized technological and epidemiological framework and the ability to monitor symptoms from the general population, including individuals who do not seek medical assistance, provided by the Influenzanet participatory surveillance platforms, are what enables the application of unsupervised algorithmic approaches such as the one presented in this work. In the next future, we will include data from virologically confirmed influenza cases as ground truth to enhance the specificity of our framework. Regarding the forecasting capabilities of the framework, approaches from existing research on participatory flu surveillance suggest that the integration of real-time official data sources with the crowdsourced digital ones [72] [73] provide better forecasting performance. In our case, the weekly integration of sufficient traditional surveillance data in the framework could lead to a near-real-time selection of the component that better represents the symptoms in the ILI syndrome circulating among the general population. Finally, the flexibility provided by the participatory surveillance platforms in terms of symptoms that can be collected from the general population enables the possibility to extend the framework to other diseases, provided that traditional surveillance data are available to train the framework.

## Supporting information

S1 Table Descriptive statistics of the Influenzanet data by country. Here, we present a few statistics regarding the available Influenzanet data for each country; (i) the number of seasons available, (ii) the average number of participants per country in a season, (iii) the average number of surveys of weekly surveys, (iv) the average percentage of surveys with at least one symptom (v) the average number of surveys per participant per season, and (vi) the average number of weeks within a single season.
(PDF)

S2 Table ILI case definitions reported by the national surveillance systems of the various countries of the Influenzanet platform. Here, we show the definitions of ILI case in the various countries of the Influenzanet platform as reported by the national surveillance systems and the WHO [17]. The table highlights the existing issue in the heterogeneity of the ILI case definition in Europe. The ECDC case definition refers to the sudden onset of symptoms with one or more systemic symptoms (fever or feverishness, malaise, headache, myalgia) plus one or more respiratory symptoms (cough, sore throat, shortness of breath).
(PDF)

S1 Fig Exploration of relative likelihood of each candidate model. The best model is the one that minimizes Eq 10, denoted as *AIC*_*c*_(*k*_*min*_), and consist of *K* syndromes. For each country we depict the relative likelihood of each candidate model (*AIC*_*c*_(*k*) − *AIC*_*c*_(*k*_*min*_)), where the *AIC*_*c*_(*k*) scores for each candidate model are compared against the AIC score of the best model *AIC*_*c*_(*k*_*min*_). We depict only models with k up to 6 and not 19 for easier visual inspection. The best model per country, with optimal number of syndromes is: (a) The Netherlands *K* = 3, (b) Belgium *K* = 3, (c) Italy *K* = 2, (d) France *K* = 4, (e) UK *K* = 3, (f) Spain *K* = 2, (g) Portugal *K* = 2, (h) Denmark *K* = 2, (i) Ireland *K* = 2. The best model is presented with dashed line.
(TIF)

S2 Fig Complete set of extracted components for the Netherlands and Belgium. Comparative Analysis of the consistency and time series of the amount *y*_*ik*_ which refers to the total number of counts associated to a syndrome *k* in day *i* for all the emerged syndromes for the Netherlands and Belgium. The blue box indicates the syndrome selected as IN_NMF by the algorithm. Right panel: contribution of each symptom to the automatically selected IN NMF component. The bars are coloured for readability purposes only.
(TIF)

S3 Fig Complete set of extracted components for Italy and France. Comparative Analysis of the consistency and time series of the amount *y*_*ik*_ which refers to the total number of counts associated to a syndrome *k* in day *i* for all the emerged syndromes for Italy and France. The blue box indicates the syndrome selected as IN_NMF by the algorithm. Note that for France the syndrome selected as IN_Gastro is the second component. Right panel: contribution of each symptom to the automatically selected IN NMF component. The bars are coloured for readability purposes only.
(TIF)

S4 Fig Complete set of extracted components for the UK and Spain. Comparative Analysis of the consistency and time series of the amount *y*_*ik*_ which refers to the total number of counts associated to a syndrome *k* in day *i* for all the emerged syndromes for UK and Spain. The blue box indicates the syndrome selected as IN_NMF by the algorithm.Right panel: contribution of each symptom to the automatically selected IN NMF component. The bars are coloured for readability purposes only.
(TIF)

S5 Fig Complete set of extracted components for Portugal, Denmark, and Ireland. Comparative Analysis of the consistency and time series of the amount *y*_*ik*_ which refers to the total number of counts associated to a syndrome *k* in the day *i* for all the emerged syndromes for Portugal, Denmark and Ireland. The blue box indicates the syndrome selected as IN_NMF by the algorithm. Note that for Denmark and Ireland we have data only for the period 2014–2017. Right panel: contribution of each symptom to the automatically selected IN NMF component. The bars are coloured for readability purposes only.
(TIF)

S6 Fig Assessment of the model’s robustness in forecasting. Left panel: qualitative comparison between the forecasted IN_NMF and the national surveillance incidence (GP) time series. To allow for easier visual inspection, the depicted IN_NMF syndromes are rescaled by a fixed factor to the respective GP incidence. On the y-axis, the sample size of the GP incidence is reported. Right panel: contribution of each symptom to the automatically selected IN_NMF component. The bars are coloured for readability purposes only.
(TIF)

S7 Fig Assessment of the model’s robustness in forecasting with randomly shuffled symptoms. Left panel: qualitative comparison between the forecasted IN_NMF—that emerges if we test our model on a **randomly shuffled** matrix of symptoms—and the national surveillance incidence (GP) time series. To allow for an easier visual inspection the depicted IN_NMF syndromes are rescaled by a fixed factor to the respective GP incidence. On the y-axis, the sample size of the GP incidence is reported. Right panel: contribution of each symptom to the automatically selected IN_NMF component. The bars are coloured for readability purposes only.
(TIF)

## Footnotes

^1^Throughout this study we employ the term *latent* as used in computer science, i.e. referring to variables that are hidden, not directly observed, but rather inferred through a mathematical model. There is no reference to the medical use of the term that usually indicates an asymptomatic infection.

^2^In the results section, we report the mean and standard deviation of the symptoms discarded per country as well as the results of the framework with respect to including the duplicate reports.
