# Supplementary material for: Unsupervised extraction of epidemic syndromes from participatory influenza surveillance self-reported symptoms
Source: PLoS Comput Biol. 2019 Apr 8;15(4):e1006173. doi: 10.1371/journal.pcbi.1006173 (PMC6472822; doi:10.1371/journal.pcbi.1006173)
Supplement: S1 Table — Here, we present a few statistics regarding the available Influenzanet data for each country; (i) the number of seasons available, (ii) the average number of participants per country in a season, (iii) the average number of surveys of weekly surveys, (iv) the average percentage of surveys with at least one symptom (v) the average number of surveys per participant per season, and (vi) the average number of weeks within a single season. (PDF) [file pcbi.1006173.s001.pdf]

Supporting Information

Table S1. Descriptive statistics of the Influenzanet data by country.

	NL	BE	IT	FR	UK	ES	PT	DK	IE
(i) Number of seasons									
	6	6	6	6	6	5	6	4	4
(ii) Average number of participants per season									
	13,450	4,209	1,830	5,757	4,676	526	1,663	1,391	406
(iii) Average number of surveys per season									
	206,987	67,420	17,807	68,567	45,543	5,894	17,852	22,782	3,220
(iv) Average percentage of surveys with symptoms									
	20%	16%	19%	20%	29%	22%	17%	18%	25%
(v) Average number of surveys per participant per season									
	15	16	9	12	9	11	10	16	8
(vi) Average number of weeks within a single season									
	21.85	21.14	18.14	18.83	19.28	18.66	18.00	22.60	20.80

Here, we present a few statistics regarding the available Influenzanet data for each country; (i) the number of seasons available, (ii) the average number of participants per country in a season, (iii) the average number of surveys of weekly surveys, (iv) the average percentage of surveys with at least one symptom (v) the average number of surveys per participant per season, and (vi) the average number of weeks within a single season.
