# Supplementary material for: Unsupervised extraction of epidemic syndromes from participatory influenza surveillance self-reported symptoms
Source: PLoS Comput Biol. 2019 Apr 8;15(4):e1006173. doi: 10.1371/journal.pcbi.1006173 (PMC6472822; doi:10.1371/journal.pcbi.1006173)
Supplement: S2 Table — Here, we show the definitions of ILI case in the various countries of the Influenzanet platform as reported by the national surveillance systems and the WHO [17]. The table highlights the existing issue in the heterogeneity of the ILI case definition in Europe. The ECDC case definition refers to the sudden onset of symptoms with one or more systemic symptoms (fever or feverishness, malaise, headache, myalgia) plus one or more respiratory symptoms (cough, sore throat, shortness of breath). (PDF) [file pcbi.1006173.s002.pdf]

Supporting Information

Table S1. ILI case definitions reported by the national surveillance systems of the various countries of the Influenzanet platform.

Country	ILI case definition	
	National Surveillance System	WHO [1]
BE	Sudden onset of fever with respiratory symptoms AND general symptoms.	
DK	Sudden onset of fever or feverishness (chills) AND any symptom of malaise, headache or muscle pain AND at least one of the following symptoms: cough, sore throat or shortness of breath.	Sudden onset of fever, muscle pain and respiratory tract symptoms.
FR	Sudden onset of fever >39°C with respiratory symptoms AND myalgia.	
IE	Sudden onset of symptoms with a temperature of 38°C or more, in the absence of any other disease, with at least two of the following: dry cough, headache, sore muscles and a sore throat.	ECDC case definition.
IT	Since 2014-2015: ECDC case definition. Before 2014-2015: Sudden onset AND fever >38°C AND at least one of the following systemic symptoms: headache, malaise, chills, sweats, fatigue; AND at least one of the following respiratory symptoms: cough, sore throat, nasal obstruction.	ECDC case definition.
NL	Pel criteria: Acute onset AND rectal temperature >38°C AND at least one of the following symptoms: cough, coryza, sore throat, frontal headache, retrosternal pain, myalgia. [2]	
PT	Sudden onset with at least one of the following systemic symptoms: fever or feverishness, malaise or weakness or prostration, headache, myalgia or general pain (muscle or joint pain); AND at least one of the following respiratory symptoms: cough, sore throat or inflammation of the nasal or pharyngeal mucosa without relevant respiratory signs (runny or blocked nose, sneezing), difficulty breathing.	ECDC case definition.
ES	Sudden onset of symptoms with at least one of the four general symptoms: fever, malaise, headache, myalgia; AND at least one of the three respiratory symptoms: cough, sore throat, dispnea; AND lack of other suspected symptoms.	ECDC case definition.
UK	No ILI case definition used.	Acute respiratory illness with physician–diagnosed fever or complaint of feverishness.

Here, we show the definitions of ILI case in the various countries of the Influenzanet platform as reported by the national surveillance systems and the WHO [1]. The table highlights the existing issue in the heterogeneity of the ILI case definition in Europe. The ECDC case definition refers to the sudden onset of symptoms with one or more systemic symptoms (fever or feverishness, malaise, headache, myalgia) plus one or more respiratory symptoms (cough, sore throat, shortness of breath).

References

1. WHO. Influenza Surveillance. Country, Territory and Area Profiles 2017.; 2017. Available from: http://www.euro.who.int/__data/assets/pdf_file/0006/321864/Influenza-surveillance-country-territory-area-profiles-2016-en.pdf?ua=1.

2. Pel J. Proefonderzoek naar de frequentie en de aetiologie van griepachtige ziekten in de winter 1963–1964. Huisarts en Wetenschap. 1965;8(321):4.
