# Supplementary figures and images for: Unsupervised extraction of epidemic syndromes from participatory influenza surveillance self-reported symptoms
Source: PLoS Comput Biol. 2019 Apr 8;15(4):e1006173. doi: 10.1371/journal.pcbi.1006173 (PMC6472822; doi:10.1371/journal.pcbi.1006173)

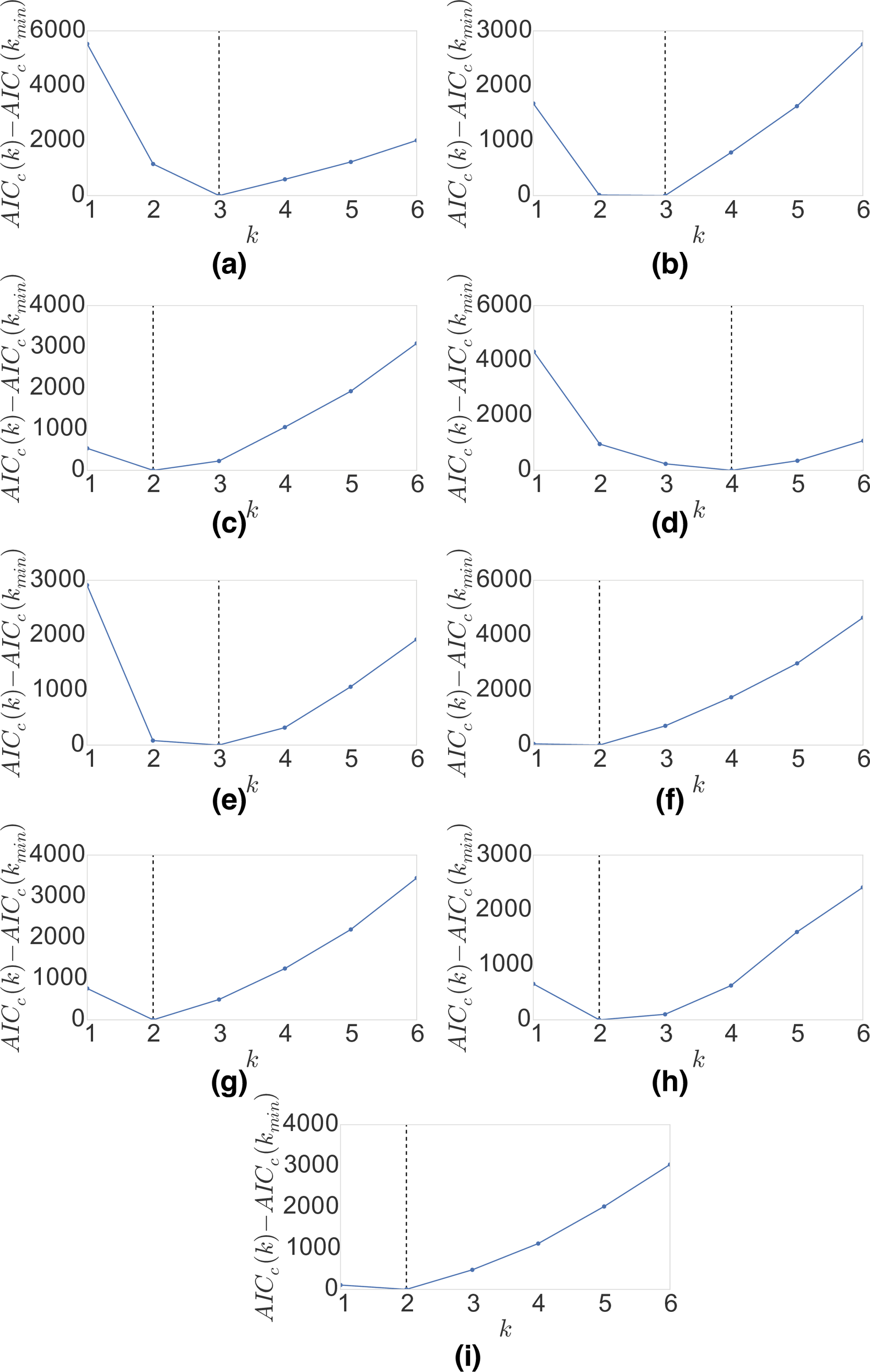

Supplement: S1 Fig — The best model is the one that minimizes Eq 10, denoted as AICc(kmin), and consist of K syndromes. For each country we depict the relative likelihood of each candidate model (AICc(k) − AICc(kmin)), where the AICc(k) scores for each candidate model are compared against the AIC score of the best model AICc(kmin). We depict only models with k up to 6 and not 19 for easier visual inspection. The best model per country, with optimal number of syndromes is: (a) The Netherlands K = 3, (b) Belgium K = 3, (c) Italy K = 2, (d) France K = 4, (e) UK K = 3, (f) Spain K = 2, (g) Portugal K = 2, (h) Denmark K = 2, (i) Ireland K = 2. The best model is presented with dashed line. (TIF) [file pcbi.1006173.s003.tif]

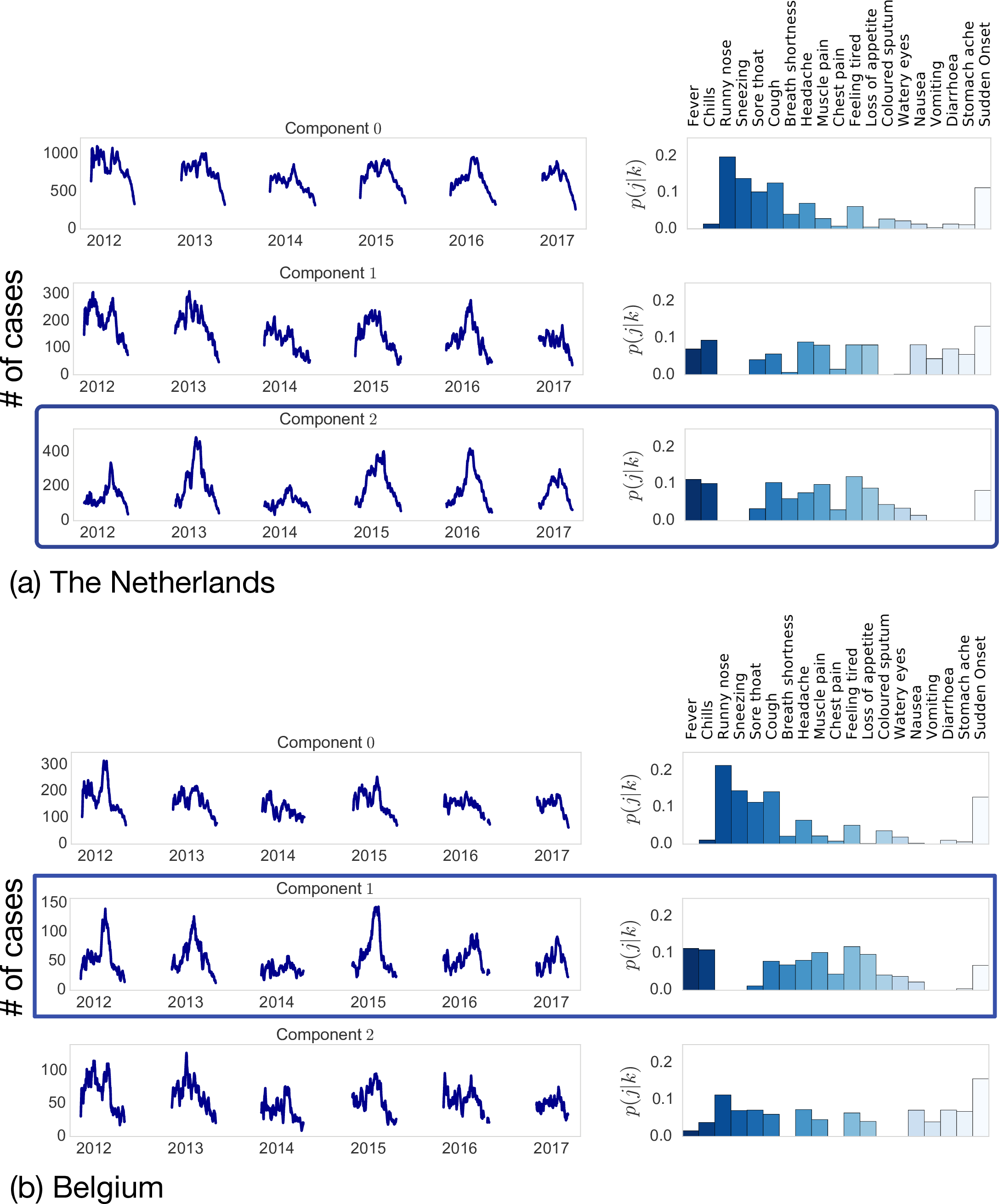

Supplement: S2 Fig — Comparative Analysis of the consistency and time series of the amount yik which refers to the total number of counts associated to a syndrome k in day i for all the emerged syndromes for the Netherlands and Belgium. The blue box indicates the syndrome selected as IN_NMF by the algorithm. Right panel: contribution of each symptom to the automatically selected IN NMF component. The bars are coloured for readability purposes only. (TIF) [file pcbi.1006173.s004.tif]

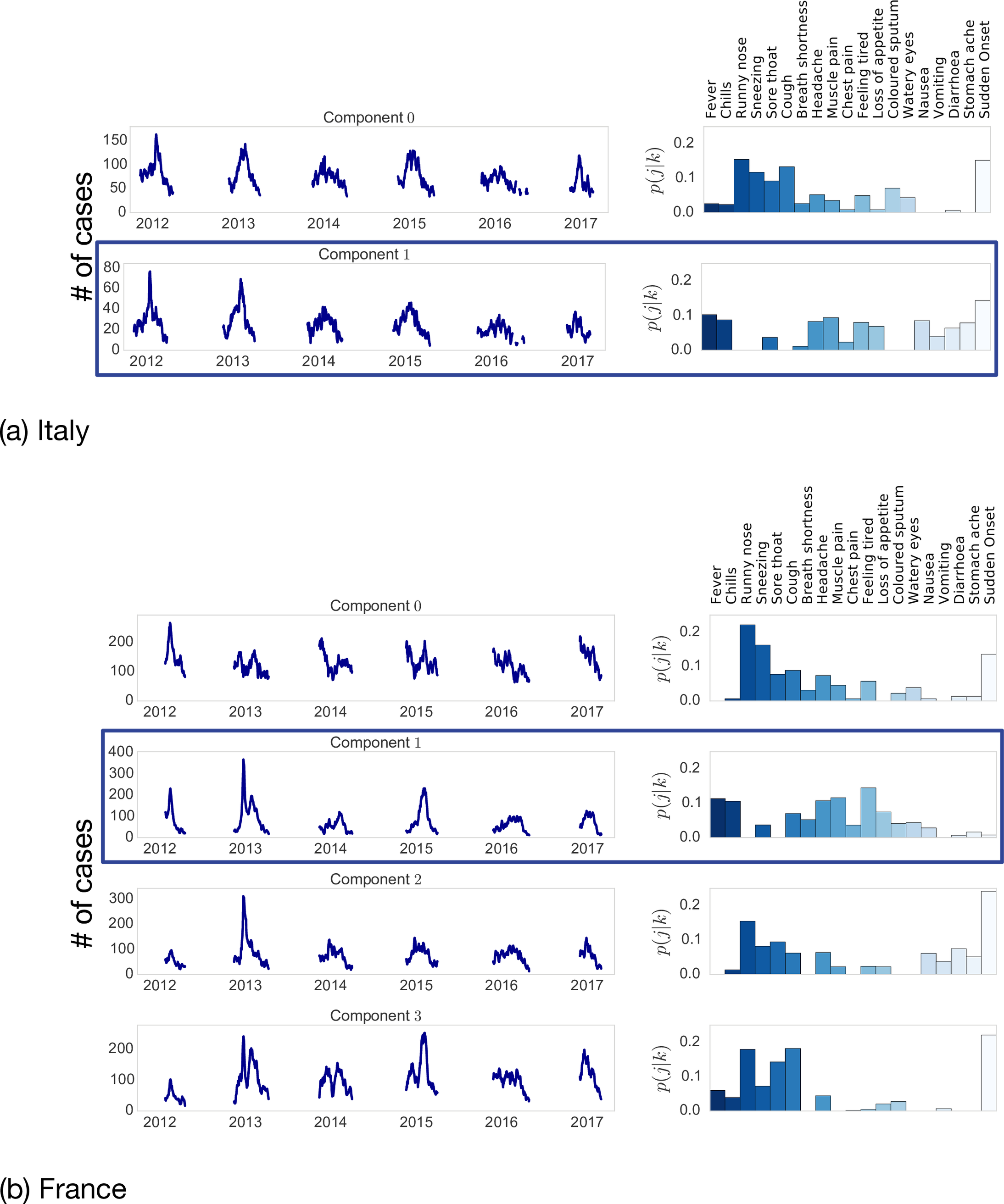

Supplement: S3 Fig — Comparative Analysis of the consistency and time series of the amount yik which refers to the total number of counts associated to a syndrome k in day i for all the emerged syndromes for Italy and France. The blue box indicates the syndrome selected as IN_NMF by the algorithm. Note that for France the syndrome selected as IN_Gastro is the second component. Right panel: contribution of each symptom to the automatically selected IN NMF component. The bars are coloured for readability purposes only. (TIF) [file pcbi.1006173.s005.tif]

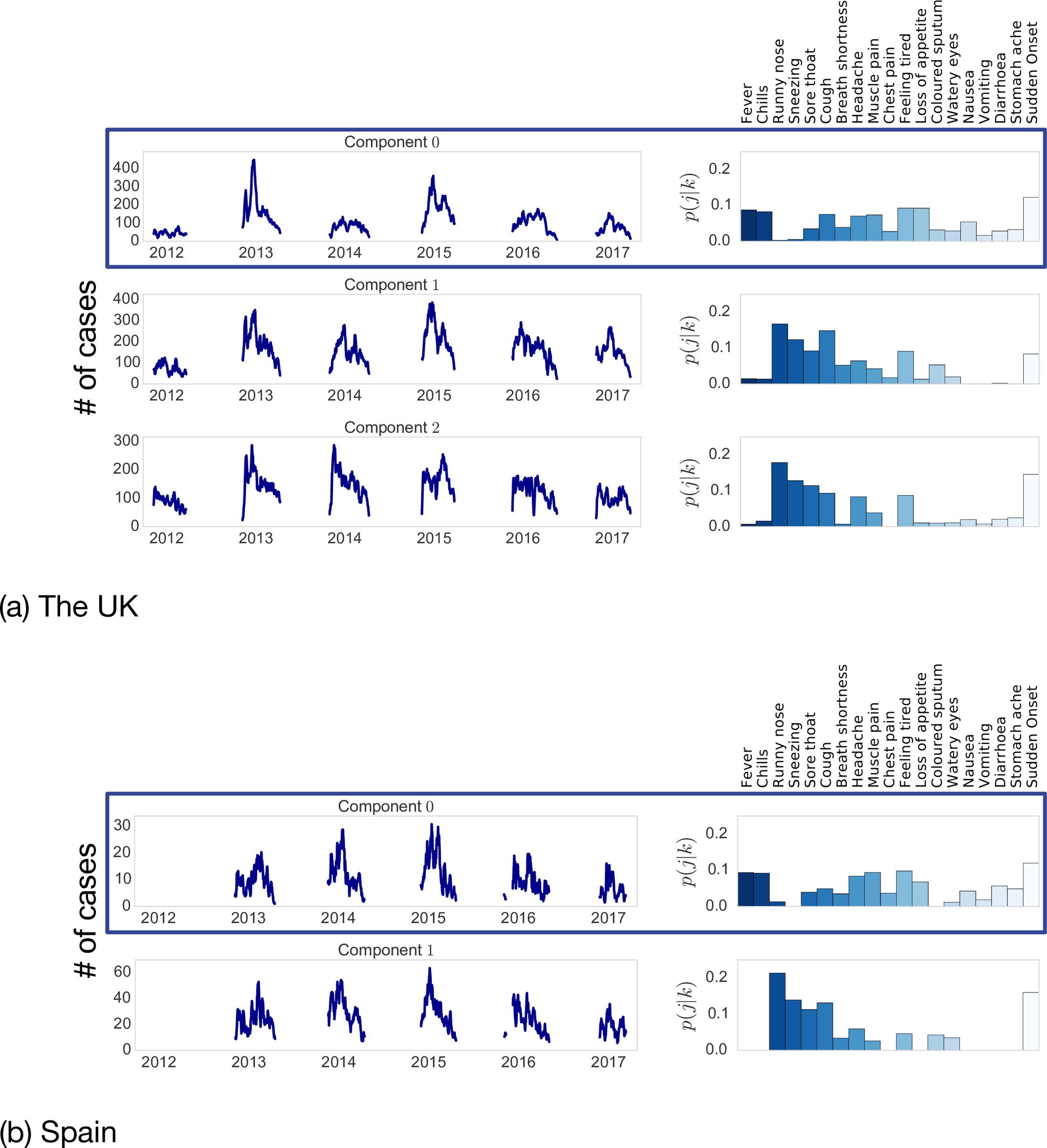

Supplement: S4 Fig — Comparative Analysis of the consistency and time series of the amount yik which refers to the total number of counts associated to a syndrome k in day i for all the emerged syndromes for UK and Spain. The blue box indicates the syndrome selected as IN_NMF by the algorithm.Right panel: contribution of each symptom to the automatically selected IN NMF component. The bars are coloured for readability purposes only. (TIF) [file pcbi.1006173.s006.tif]

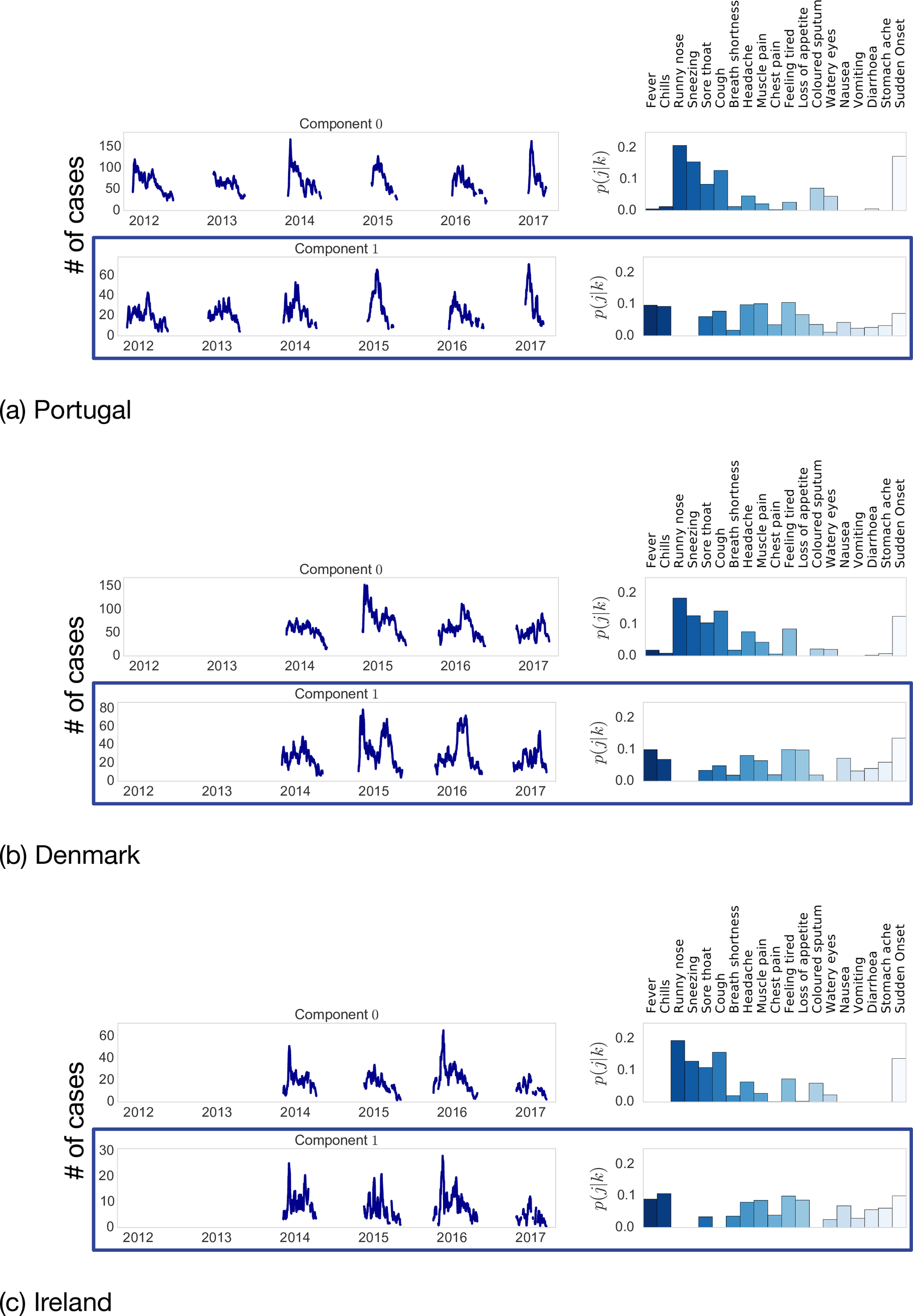

Supplement: S5 Fig — Comparative Analysis of the consistency and time series of the amount yik which refers to the total number of counts associated to a syndrome k in the day i for all the emerged syndromes for Portugal, Denmark and Ireland. The blue box indicates the syndrome selected as IN_NMF by the algorithm. Note that for Denmark and Ireland we have data only for the period 2014–2017. Right panel: contribution of each symptom to the automatically selected IN NMF component. The bars are coloured for readability purposes only. (TIF) [file pcbi.1006173.s007.tif]

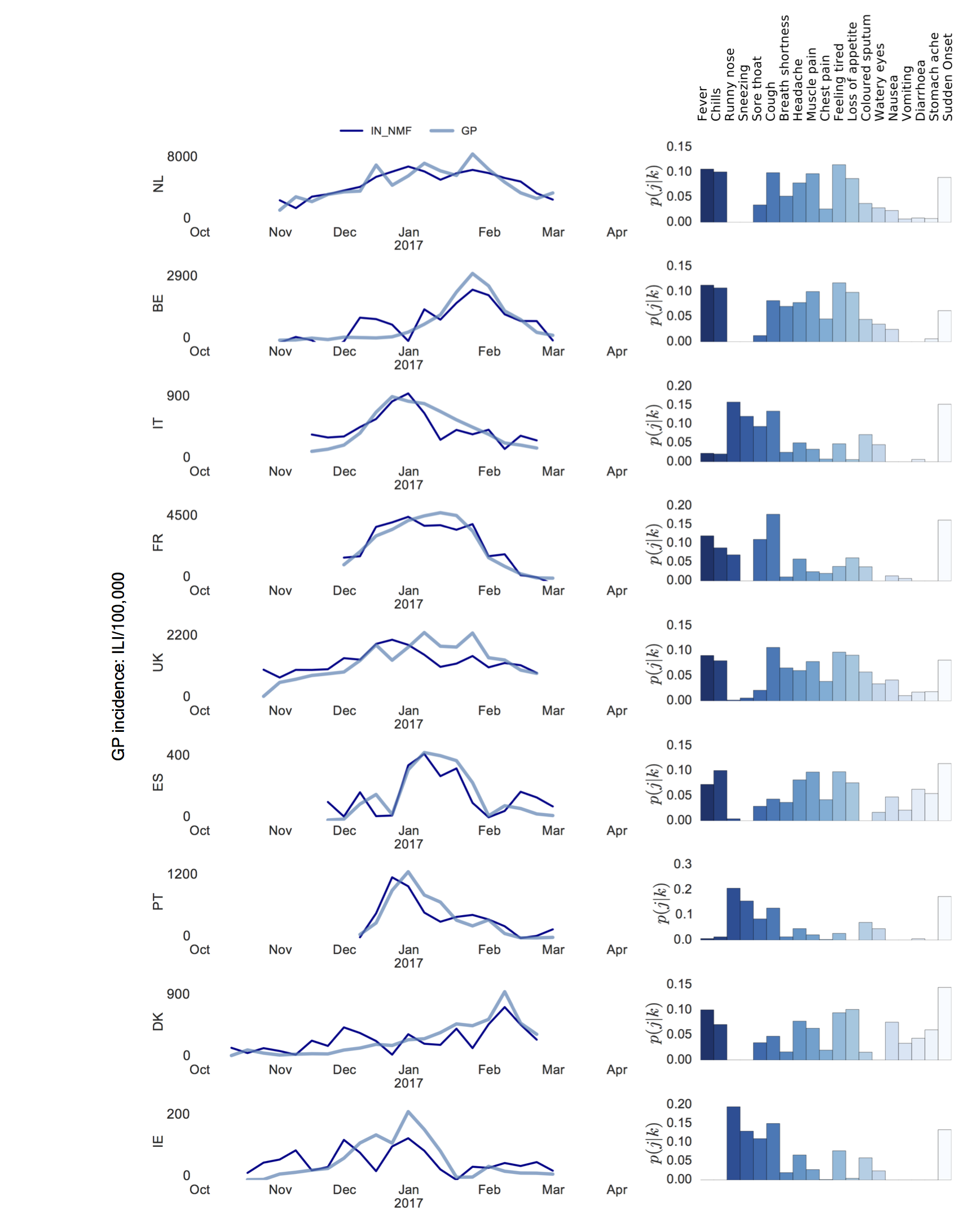

Supplement: S6 Fig — Left panel: qualitative comparison between the forecasted IN_NMF and the national surveillance incidence (GP) time series. To allow for easier visual inspection, the depicted IN_NMF syndromes are rescaled by a fixed factor to the respective GP incidence. On the y-axis, the sample size of the GP incidence is reported. Right panel: contribution of each symptom to the automatically selected IN_NMF component. The bars are coloured for readability purposes only. (TIF) [file pcbi.1006173.s008.tif]

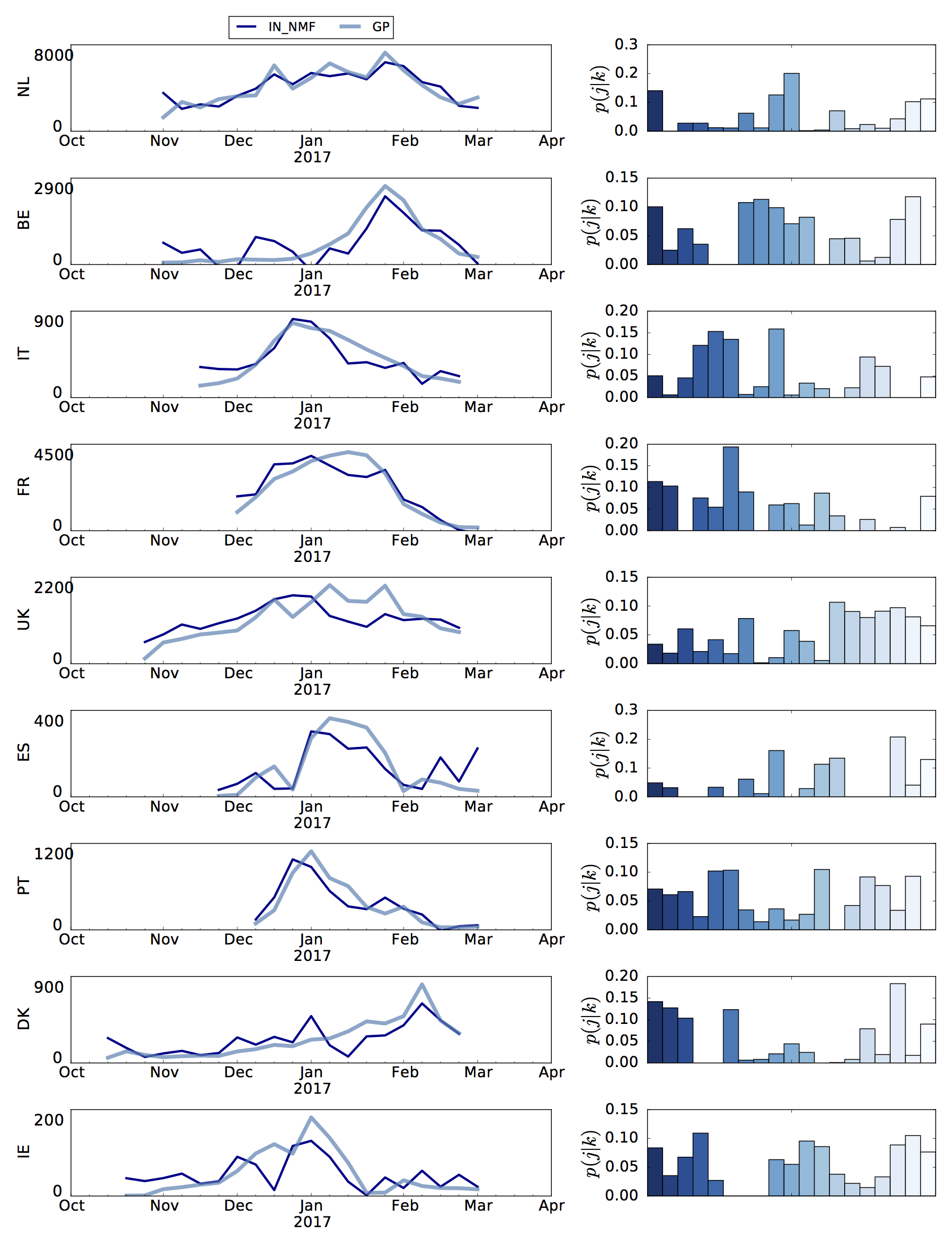

Supplement: S7 Fig — Left panel: qualitative comparison between the forecasted IN_NMF—that emerges if we test our model on a randomly shuffled matrix of symptoms—and the national surveillance incidence (GP) time series. To allow for an easier visual inspection the depicted IN_NMF syndromes are rescaled by a fixed factor to the respective GP incidence. On the y-axis, the sample size of the GP incidence is reported. Right panel: contribution of each symptom to the automatically selected IN_NMF component. The bars are coloured for readability purposes only. (TIF) [file pcbi.1006173.s009.tif]
